# Reactivity of the contact tests (patch test) in elderly patients compared to non-elderly at the allergy clinic of the Policlínica Geral do Rio de Janeiro

**DOI:** 10.1186/1939-4551-8-S1-A202

**Published:** 2015-04-08

**Authors:** Luiz Carlos Arcanjo

**Affiliations:** 1 Policlinica Geral Do Rio De Janeiro, Brazil

## Background

Senility often brings with it a series of changes in the immune system (immunosenescence) which may affect, among other areas, the lymphocyte-mediated response. The objective of this research was to evaluate the influence of immunosenescence on cellular response in patients submitted to contact patch test at the Allergy Clinic of Policlínica Geral do Rio de Janeiro.

## Methods

Retrospective analysis of patients submitted to contact test from january 2012 to june 2014, comparing the group of elderly (over 60 years) and the non-elderly group. The results were statistically analyzed using the SPSS stastitic software.

## Results

Two hundred and thirty six patients were submitted to contact test. Seventy five (31.8%) elderly (> 60 years) and 161 (68.2%) non-elderly (10-59 years). Among elderly, 46 (62.7%) had positive tests, and 29 (37.3%) negative tests. In non-elderly , 112 (69.6%) had positive and 49 (30.4%) negative tests. Despite the difference in positivity between elderly and non-elderly patients, the result was not statistically significant by Chi-square test (p = 0.366). The most frequent sensitizers in the elderly group were fragance-mix (31,9%), Euxyl K – 400 (23,4%), cobalt chloride (23,4%), imidazole derivates (21,3%) and nickel sulfate (21,3%); in the non-elderly group the most frequent contact allergens were nickel sulfate (42,9%), cobalto chloride (42,9%), fragance-mix (26,8%) and imidazole derivate (16,1%). In elderly group, four pacients were over 80 years old, including two positive tests.

## Conclusions

The immune response to contactants seems not to be affected by immunosenescence. Therefore, contact tests should be considered in this age group.

